# Transgenerational Response to Nitrogen Deprivation in *Arabidopsis thaliana*

**DOI:** 10.3390/ijms20225587

**Published:** 2019-11-08

**Authors:** Monica Massaro, Emanuele De Paoli, Nicola Tomasi, Michele Morgante, Roberto Pinton, Laura Zanin

**Affiliations:** Dipartimento di Scienze Agroalimentari, Ambientali e Animali, University of Udine, via delle Scienze 206, I-33100 Udine, Italy; monica.massaro@uniud.it (M.M.); emanuele.depaoli@uniud.it (E.D.P.); nicola.tomasi@uniud.it (N.T.); michele.morgante@uniud.it (M.M.); roberto.pinton@uniud.it (R.P.)

**Keywords:** nitrate transporter, nitrogen deficiency, RNAseq, root uptake, transcriptomic profile

## Abstract

Nitrogen (N) deficiency is one of the major stresses that crops are exposed to. It is plausible to suppose that a stress condition can induce a memory in plants that might prime the following generations. Here, an experimental setup that considered four successive generations of N-sufficient and N-limited Arabidopsis was used to evaluate the existence of a transgenerational memory. The results demonstrated that the ability to take up high amounts of nitrate is induced more quickly as a result of multigenerational stress exposure. This behavior was paralleled by changes in the expression of nitrate responsive genes. RNAseq analyses revealed the enduring modulation of genes in downstream generations, despite the lack of stress stimulus in these plants. The modulation of signaling and transcription factors, such as *NIGTs*, *NFYA* and *CIPK23* might indicate that there is a complex network operating to maintain the expression of N-responsive genes, such as *NRT2.1*, *NIA1* and *NIR*. This behavior indicates a rapid acclimation of plants to changes in N availability. Indeed, when fourth generation plants were exposed to N limitation, they showed a rapid induction of N-deficiency responses. This suggests the possible involvement of a transgenerational memory in Arabidopsis that allows plants to adapt efficiently to the environment and this gives an edge to the next generation that presumably will grow in similar stressful conditions.

## 1. Introduction

Plants are usually subject to large seasonal fluctuations in light, temperature, water and nutrients’ availability, often to levels that are sub-optimal for plant growth, thus, they are continuously exposed to environmental stresses [1]. Considering the predicted demographic increase in the next 30 years and the current soil consumption rate, it is important to find new strategies to guarantee crop productivity and to maintain high growth under sub-optimal environmental conditions. It has been estimated that around 60% of cultivated soils lead to nutritional disorders in crops (deficiency or toxicity impairments) [2]. Of these nutritional stresses, nitrogen (N) deficiency is one of the most limiting factors for plant growth in both natural and agricultural ecosystems. Lack of macronutrients, like N, phosphorus and potassium, strongly impairs plant growth; however, plants have developed specific mechanisms to activate physiological and molecular responses to counteract the low nutrient availability in the soil [1,3,4]. Indeed, when deprived of an external N source, plants increase their ability to take up N [4,5,6].

Nitrogen is essential for adequate plant growth and consists of several primary metabolites (such as amino acids, nucleic acids, pigments) as well as secondary metabolites (such as amines, phytohormones, alkaloids). Symptoms of N deficiency include impaired plant development, leaf chlorosis and reduced quality and quantity of crop productivity. To date, the N use efficiency (NUE) of crops is very low (around 30%–35% for cereals) [7]. Hence it is crucial to develop new strategies to increase NUE. 

Compelling evidence from field observations and recent experimentation [8] indicate that plants can preserve information from past environmental events and use such memories, in the form of molecular records, to support their response when these events occur again. A priming effect has been hypothesized, through which a previous exposure to a stress provides plants with a stronger capacity for resilience to counteract future stress events. Primed plants show either faster and/or stronger activation of the various defense responses that are induced following attacks by either pathogens or herbivores, or in response to abiotic stresses [9]. For instance, in Arabidopsis, a previous exposure to either osmotic or oxidative stress can markedly alter subsequent osmotic-stress-induced Ca^2+^ responses, indeed the nature of the alterations in Ca^2+^ response depends on the identity and severity of the previous stress, suggesting that there is an imprint of previous stresses [10]. Other evidence highlights that, in Arabidopsis, drought signals are converted into modulations of gene expression [11] and such changes in expression are commonly accompanied by variations in the chromatin status [12,13]. 

Based on these considerations, it is reasonable to expect that some epigenetic mechanisms might also be involved in responses to nutritional stress; through this mechanism, the responses could be transmitted to their progenies, stabilizing stress-dependent gene expression changes, and thus enhancing the ability of the plants to acquire nutrients. Drought environments are also a common, stressful event for plants and involve changes in the methylation status [14]. Moreover, in Arabidopsis, heat stress induces the transcription of the retrotransposon *ONSEN* and the transgenerational retrotransposition of this element involves an epigenetic mechanism [15]. In favor of this hypothesis, evident heritable epigenetic modifications induced by different nutritional stresses in mammals have already been reported [16,17,18,19]. In spite of these promising observations and the convincing proof of the involvement of epigenetic events in the transgenerational memory to salt stress in Arabidopsis [20], the role of epigenetics in transgenerational stress memory in plants is still controversial. 

A priming memory for a specific stress has a beneficial effect on plant fitness because it supports an enhanced or more rapid response to the stress when it reappears [21]. Responses to abiotic stresses are known as acclimation or hardening; these responses can also be reinforced by priming treatments. Priming can be elicited by exogenous application of chemical treatments as well as by exposure to stress signals themselves [22,23]. Based on these observations, it can be assumed that multiple exposures to stress enables plants to respond to a new stress with quicker adaptive changes in gene expression patterns compared with not previously exposed plants [24].

The aim of this work was the characterization of the putative transgenerational response of *Arabidopsis thaliana* plants to limiting N conditions, which were evaluated at the morphological, physiological and molecular levels. The occurrence of a potential transgenerational memory was evaluated in four successive generations of plants that were exposed to different regimes of N availability. By using this approach, we aimed to characterize the potential transgenerational response to N deficiency, as revealed by reinforced tolerance to stress, enhanced capabilities to modulate N-uptake mechanisms, morphological adaptation and gene regulation of nutritional pathways, to obtain a comprehensive overview of the plasticity of plant species to adapt to recurring nutritional stress conditions.

## 2. Results

**Figure 1:**
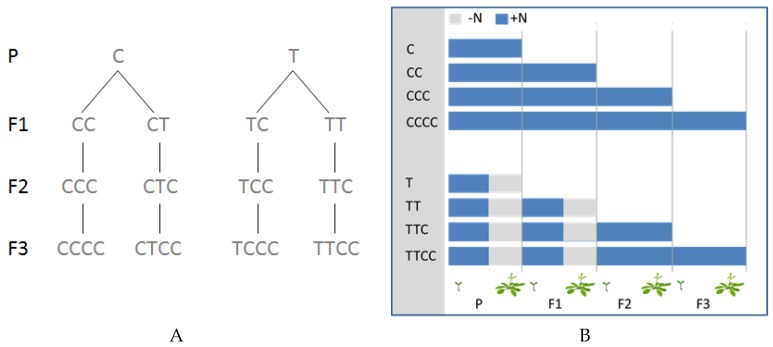
Experimental set up of four generations (P–F3) of Arabidopsis plants (**a**). In the right panel a schematic representation of the thesis used for RNAseq experiments (**b**). Blue bars refer to N-sufficient period (+N), grey bars refer to N-deficient treatment (one week long, −N). The thesis C, CC, CCC, CCCC refer to N-sufficient plants of first, second, third and fourth generations (P, F1, F2, and F3), respectively; T and TT refer to N-limited plants during P or P and F1, respectively; CTC, TCC, TTC and CTCC, TCCC, TTCC refer to N-sufficient plants during the current generation that were exposed to N limiting conditions in the previous generation/s.

In this study, four generations of Arabidopsis plants were grown under hydroponic conditions with two different N supply regimes and the subsistence of a priming effect inherited by their progeny was assessed (Figure 1). Different batches of plants were exposed to N limiting conditions in none, either or both the two initial generations. In the following generations, plants from both the N-limited and the corresponding control plants (N-sufficient plants) were maintained under N sufficiency to evaluate any persisting effects triggered by the initial stress exposure(s). 

Morphological data indicated that in N-limited plants of the first and second generation (called T and TT hereafter, respectively), the low N availability induced an increase in root fresh weight (FW) while biomass accumulation was reduced at the leaf level in comparison to N-sufficient plants (Figure S1). As expected, N-limited plants (T, TT) accumulated less N than N-sufficient ones (Figure S2). Indeed, the CHN analyses indicated a reduction in N content in first- and second-generation plants grown under N-limiting condition (T, CT and TT) in comparison to N-sufficient plants (C, CC, TC) leading to a high carbon to N ratio in TT plants. On the other hand, no significant changes in N and in the carbon to N ratio (also in FW and root volume) were present among plants of the third generation after growth in N-sufficient condition (CCC, TCC, CTC, TTC), irrespective of the N-treatment applied in previous generations (Figures S1 and S2). 

**Figure 2:**
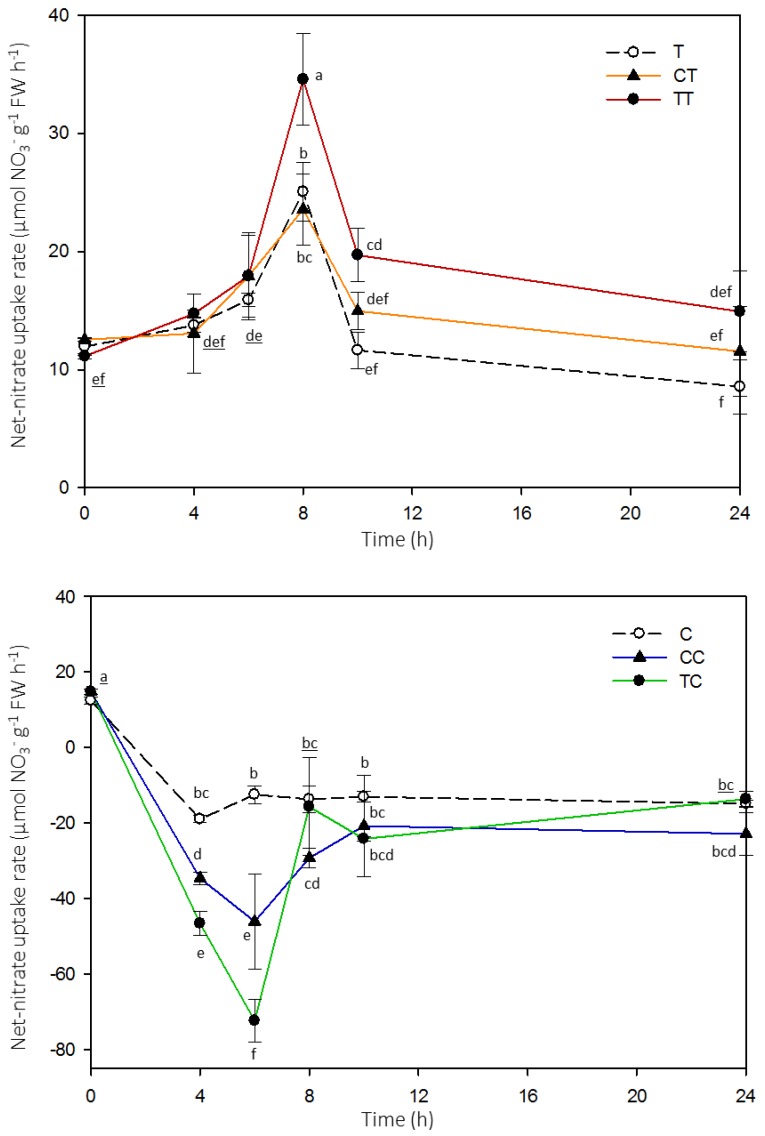
Time-course of high-affinity net-nitrate uptake rate in Arabidopsis roots. During the time span of the experiment (24 h), Arabidopsis plants were exposed to a nutrient solution containing 1 mM nitrate. After 0, 4, 6, 8, 10 or 24 h, groups of six plants from each treatment were transferred into the assay solution (0.5 mM CaSO_4_ containing 100 μM nitrate, up to 10 min). The values are means ± SD, small letters refer to statistical significance; underlined letters refer to statistical significance of two overlapping points (one-way ANOVA, Student-Newman-Keuls method, *N* = 3, *p*-value < 0.05). FW, fresh weight.

At the physiological level, the data indicated that the net nitrate uptake rate into the root was induced by the presence of nitrate in the external solution, and this response occurred only on N-limited plants (Figure 2). Indeed, when Arabidopsis plants were grown in a N-containing solution, they did not promote the high-affinity influx of nitrate but rather stimulated nitrate efflux from roots. This pattern was visible in first and second generation plants. Moreover, TT plants that had been subjected to limited availability of N in the first two generations showed the greatest ability to take up nitrate, higher than CT plants (which were subjected to N deficiency condition only in the second generation) and T plants.

**Figure 3:**
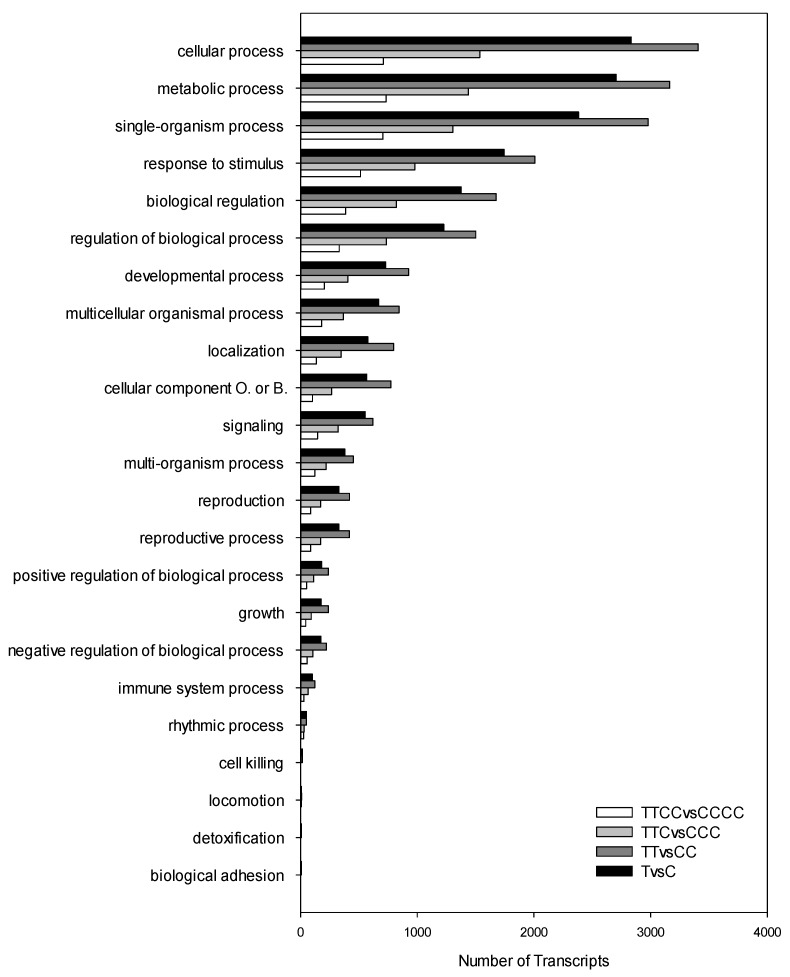
Enrichment analyses of biological process categories (gene ontology (GO) terms) of differentially modulated transcripts in four comparisons. Black bars refer to T vs. C; dark grey bars refer to TT vs. CC; light grey bars refer to TTC vs. CCC; white bars refer to TTCC vs. CCCC (*N* = 3, *q*-value < 0.05).

Prompted by such results at the physiological level, we performed comparative RNAseq analyses that highlighted differences in root transcriptomic profiles among generations. Transcriptomic analyses were performed on plants grown under N-sufficient conditions (C, CC, CCC, CCCC) or under N-limiting conditions (T, TT), or on N-sufficient plants from the third and fourth generation that were twice exposed to limited availability of N in their previous generations (TTC, TTCC). In Table 1, the total amounts of differentially modulated transcripts are shown: 5709, 7015, 3075 and 1443 transcripts were significantly modulated in T vs. C, TT vs. CC, TTC vs. CCC and TTCC vs. CCCC comparisons, respectively. Analyses of gene ontology (GO) enrichment allowed the identification of the biological processes that were the most influenced by N-limiting treatments (Figure 3; Tables S1–S3). 

The most enriched categories were: “cellular process” (GO:0009987), “metabolic process” (GO:0008152), “single organism process” (GO:0044699), “response to stimuli” (GO:0050896), “biological regulation” (GO:0065007), “regulation of biological process” (GO:0050789), and they were significantly enriched for all comparisons (Table S1). In each category, the number of modulated transcripts was the highest in TT vs. CC, followed by T vs. C, TTC vs. CCC, and TTCC vs. CCCC.

**Figure 4:**
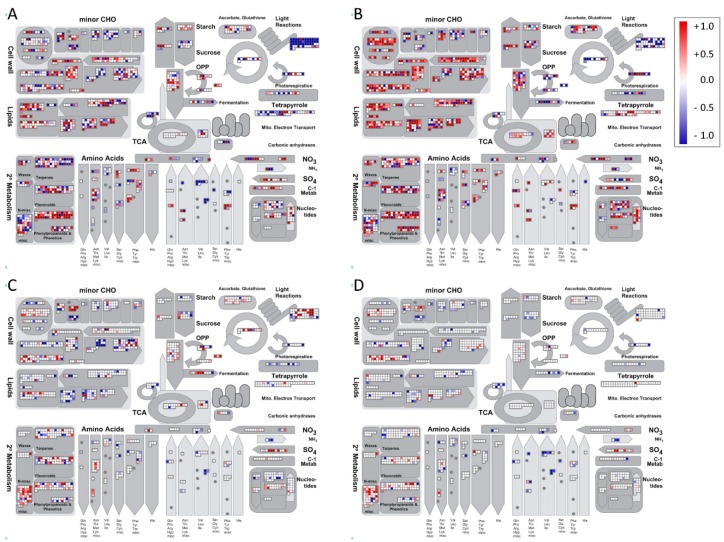
Metabolic overviews referred to four comparisons: T vs. C (panel **A**), TT vs. CC (panel **B**), TTC vs. CCC (panel **C**), TTCC vs. CCCC (panel **D**). The color scale red-blue illustrates Log_2_FC values, the red color refers to upregulated transcripts, the blue color refers to downregulated ones (*N* = 3, *q*-value < 0.05).

Differentially expressed transcripts were mapped on a schematic representation of plant cell metabolism using MapMan software (Figure 4). The comparison TT vs. CC mapped the highest number of modulated transcripts than the other comparisons and were mainly related to primary metabolism (such as glycolysis, TCA cycle, N assimilation) as well as secondary metabolism and cell wall/lipid modifications. 

**Figure 5:**
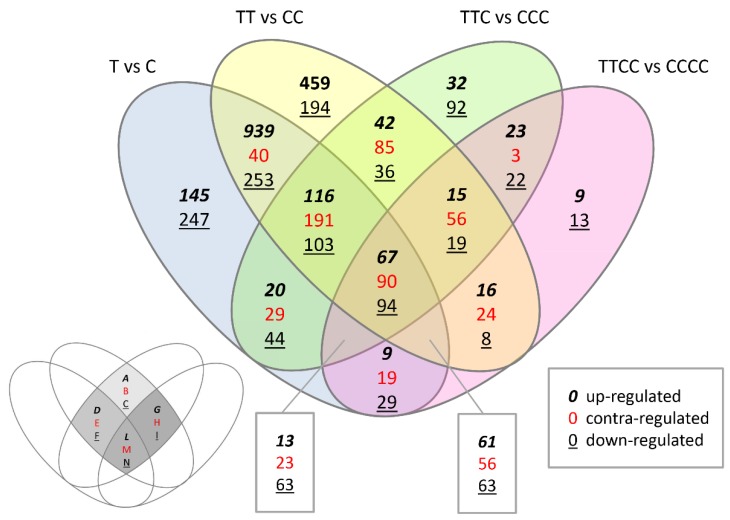
Venn diagram of differentially modulated transcripts in four comparisons: T vs. C, TT vs. CC, TTC vs. CCC, TTCC vs. CCCC. In the bottom left panel, a schematic representation of Venn diagram region names as reported in Table 2 and Table 3 referring to gene modulation shared by TT vs. CC and TTC vs. CCC. All significantly modulated transcripts were filtered using Log_2_FC ≥ |1.00| as threshold in at least one of the four comparisons (*N* = 3, *q*-value < 0.05).

Modulated transcripts with a Log_2_FC ≥|1.00| were clustered by Venn diagram. Results showed that some transcripts modulated by N deficiency treatment in the first and/or second generation were still modulated in the third and fourth generation (341 transcripts, according to Venn diagram regions G to N of Figure 5) although those plants were never exposed to N deficiency during the current generation (TTC) or during the last two generations (TTCC).

A particular attention has been paid to twelve regions of modulated transcripts of the Venn diagram (Venn diagram regions A to N, see Figure 5), that correspond to the intersection of modulated transcripts by TT vs. CC and by TTC vs. CCC (TT vs. CC ∩ TTC vs. CCC; Table 2 and Table 3). These subgroups of transcripts allowed us to uncover some genes that were strongly suggestive of a memory response, since their modulation was caused by N deficiency stress in the previous generation/s and remained modulated in the following generation/s. Among these transcripts some of them are known to code for N transporters or enzymes involved in N assimilation, such as *NRT1.1*, *NRT2.1*, *AMT1;1*, *NIA1*, *NIR1*, *GLN1;1*, *GDH1* and *GDH2*. Other transcripts code for transcription factors, such as LBD, bZIP, MYB, bHLH, WRKY, NIGTs or regulatory protein, such as CIPK23, known to be involved in the regulation of N acquisition process.

**Figure 6:**
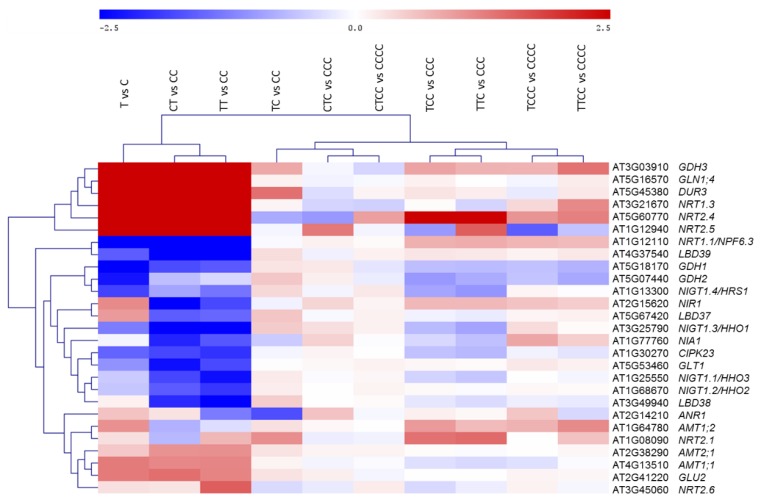
Gene expression analyses of some genes related to N response by real-time RT-PCR experiments. The color scale red-blue refers to Log_2_FC values of differentially modulated transcripts, red refers to upregulated transcripts, blue refers to downregulated ones (*N* = 3). *AMTs*, ammonium transporters; *ANR*, MADS box transcription factor; *CIPK*, CBL-interacting protein; *DUR3*, urea transporter; *GDH*, glutamate dehydrogenase; *GLN*, glutamine synthetase; *GLT1*, NADH-dependent glutamate synthase 1; *GLU2*, ferredoxin-dependent glutamate synthase 2; *LBD*, LOB domain-containing protein; *NIA*, nitrate reductase; *NIGTs*, nitrate-inducible GARP-type transcriptional repressors; *NRTs*, nitrate transporters.

Several transcripts known to be involved in N acquisition and metabolism were also analyzed via real-time RT-PCR (Figure 6). Gene expression analyses were performed on every root sample collected as indicated in the experimental set up (Figure 1a). These quantification confirmed the pattern observed by RNAseq analyses, in particular the sample tree of clustering analysis indicated that the expression of N-responsive genes was highly modulated in response to the N-limiting treatment/s (e.g., the up-regulation of genes coding for N-transporters: some NRTs, AMTs and DUR3 in T vs. C, TT vs. CC and CT vs. CC comparisons) while the other comparisons clustered together indicating a similar trend of gene expression among N-sufficient conditions (TC vs. CC; CTC-, TCC-, and TTC vs. CCC; TCCC-, TTCC-, and CTCC vs. CCCC). It is interesting to note that the intensity of gene expression modulation was higher in TTC vs. CCC than in CTC vs. CCC and the same held true for TTCC vs. CCCC in comparison to CTCC vs. CCCC (Figure 6).

**Figure 7:**
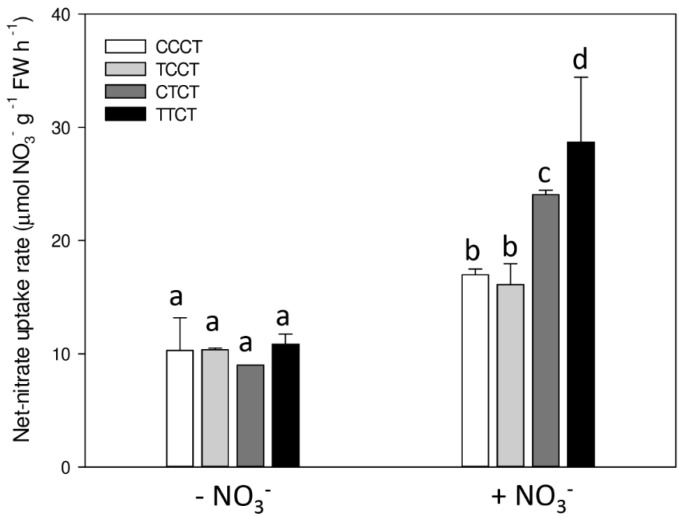
Net-uptake rate of nitrate into Arabidopsis roots of fourth generation plants. To evaluate the potential capability of Arabidopsis roots to take up nitrate, all plants were exposed to one week of N-limiting conditions before the assay. The day of the experiment, half the plants were maintained in N-free nutrient solution (−NO_3_^−^), while the other half were transferred to a nutrient solution containing 1 mM nitrate (+NO_3_^−^) and maintained in these solutions for 8 h. Six plants from each treatment were transferred to the assay solution (0.5 mM CaSO_4_ containing 100 μM nitrate) and incubated up to 10 min. The values are means +SD, small letters refer to statistical significance (Student-Newman-Keuls Method) method, *N* = 3, *p*-value < 0.05). FW, fresh weight.

To evaluate the ability of fourth generation plants to be responsive to N fluctuation in the external media, N-sufficient plants of the fourth generation were exposed to one week of N limiting conditions (CCCT, TCCT, CTCT and TTCT plants) and the net nitrate uptake rate by high affinity transport system was measured in roots after an induction of 8 h with 1 mM nitrate (corresponding to the peak of the curve as presented in Figure 2). Data show that TTCT plants exhibited the highest uptake rate of nitrate (approx. 28 µmol NO_3_^−^ g^−1^ FW h^−1^) while lower values were obtained by CTCT (approx. 24 µmol NO_3_^−^ g^−1^ FW h^−1^) and by TCCT and CCCT (both treatments were approx. 16 µmol NO_3_^−^ g^−1^ FW h^−1^; Figure 7).

## 3. Discussion 

Higher plants have evolved different mechanisms to ensure their adaptation to environmental changes [25]. It has been speculated that the ability of plants to be responsive to environmental stimuli can also be inherited by successive generations of these plants [25]. To date, the mechanisms involved in this process are still unknown. However, there is compelling evidence for the involvement of regulatory processes on gene transcription that might be mediated by epigenetic events (e.g., changes in the chromatin status, DNA methylation), as reported for the regulation of vernalization response, genomic imprinting, defense against pathogens and stress responses [26,27,28,29,30,31]. 

In this work, the morphological, physiological and transcriptomic responses of Arabidopsis plants to low N availability were investigated in four successive generations to understand if a period of N deficiency can influence the response of their progenies. 

Morphological observations of roots and leaves indicated an increase in root-to-shoot biomass in N-limited plants (confirming the evidence in [32]). However, this behavior was not followed by an alteration in the plant biomass in the following generations (when grown with an adequate supply of N; Figure S1). 

Beside morphological changes, Arabidopsis plants showed differences in their capability to take up nitrate using the high-affinity transport system (Figure 2). In plants, nitrate acquisition is a process induced by nitrate itself, and it is retroregulated by ammonium and other N-containing compounds. Moreover, this process is mediated by transcriptional and post-transcriptional events, acting directly on genes and proteins involved in the uptake and assimilation of N [33]. In recent years, studies have demonstrated the role of the dual-affinity nitrate transporter NRT1.1 in the activation of the inducible nitrate transport system [34], which consists mainly of the high-affinity transport mediated by NRT2.1 and NRT2.2, and by partner protein NAR2.1. In this work, the exposure of plants during the first and/or second generations to low-N availability (T, CT and TT plants) stimulated the uptake rate of nitrate into roots (Figure 2). The maximum net uptake rate of nitrate was obtained by roots of TT plants, which were subjected to N-limitation in both the first and second generation, suggesting an overstimulation in plants that have already overcome the same N-limiting condition in previous generations. Moreover, this result was further confirmed when the N-limiting conditions were reintroduced in the fourth generation (Figure 7). These results provide physiological evidence for the existence of a memory over multiple generations after a period of N limitation. As reviewed by Kinoshita and Seki [8], the capability of plants to improve their acclimation to the environmental conditions is also strengthened in plants that are re-exposed to drought stress [24], and was linked to changes at the transcriptional level. 

The RNAseq analyses of four generations of plants allowed the identification of transcriptional changes that could contribute to the observed priming effect. As expected, N-limitation induced changes in the transcriptomic profiles in comparison to N-sufficient plants (Table 1) [35,36]. It is interesting to note that in the third and fourth generations, TTC and TTCC plants showed significant changes at the transcriptomic level compared to CCC and CCCC plants, respectively, despite all of them being exposed to a sufficient N regime (in the last two generations). This observation suggests that N-limiting conditions occurring in previous generations, stimulated their progeny to maintain the modulation of their transcriptional response. This modulation involved twice as many genes in TTC than in TTCC, suggesting that the number of N-sufficient generations could be relevant for the priming effect decay.

Some transcripts responsive to N limitation were found modulated by T and TT plants and were maintained modulated in the third generation (TTC plants, Table 2) or in the third and fourth generations (TTC and TTCC plants, Table 3). Among these transcripts, some are known to be directly involved in the transport and assimilation of N, such as *NRT1.1*, *NRT2.1*, *AMT1;1*, *GLN1;1*, *NIA1*, *NIR1*, *GDH1* and *GDH2*. Moreover, other transcripts are known to be involved in nitrate signaling, such as *CIPK23, NIGT1;1*, *NIGT1;3*, *NIGT1;4* along with other transcription factors (*MYBs*, *WRKYs*, *bHLHs*).

In particular, CIPK23 is responsible for NRT1.1 phosphorylation; after this modification, NRT1.1 acts as a high affinity nitrate transporter [37] and sensor for the induction of genes related to N-deficiency adaptation, such as *NRT2.1*. This latter transcript was found to be upregulated by the roots of TT, TTC and TTCC plants, indicating that Arabidopsis plants induced the high affinity transport system of nitrate during the second period of N-limitation and kept it induced for two generations after these nutritional stresses, even if at that time, N was present in the growth medium. 

Plant response to low N availability involves a complex network of transcription factors, including NFYA, which putatively activates the expression of high affinity nitrate transporters [38]. Thus, *NFYA* upregulation could be consistent with the induction of *NRT2.1* in TT and TTC plants. Moreover, TTC plants also exhibited a concomitant downregulation of *CIPK23* and the upregulation of *NRT1.1*.

In the non-phosphorylated state, the NRT1.1 works as a low affinity nitrate transporter, mediating the acquisition when the anion is present at high concentrations in the external solution (above 0.5 mM nitrate). Therefore, the downregulation of *CIPK23* might suggest that, especially in TTC plants, both mechanisms for the acquisition of nitrate were preserved and active: high-affinity transporters (NRT2.1) and low-affinity ones (unphosphorylated NRT1.1). 

In recent years, the involvement of GARP transcription factors to regulate the nitrate response in plants has been investigated [39]. In particular, NIGT1;4 (HRS1) together with its homolog NIGT1;3 (HHO1), acts downstream to the NRT1.1 transceptor to repress primary root growth under phosphorus deficiency. As expected, the transcription factors *NIGTs* were downregulated by N-limiting conditions, and they remained downregulated in the following generations, even if N was present in the external media. In the literature, NIGTs are reported to repress nitrate responsive genes [40], therefore their downregulation in TTC and TTCC plants might prolong the expression of N-responsive genes, such as *NRT2.1*, *NIA1* and *NIR* [41].

Concerning N assimilation, the *NIA1*, *NIR*, *GLN1;1*, *GDH1* and *GDH2* genes were found to be modulated by TTC and TTCC as well. In these generations, the overall upregulation of N-assimilatory genes suggests that the nitrate transport was sustained by a concomitant metabolic activity. 

Overall, these data suggest the existence of a transgenerational memory that causes transcriptional modulation of N-responsive genes. The expression levels of approx. 30 N-responsive genes (not only transcription factors, but also coding for N transporters, metabolic enzymes and associated proteins) in plants of the third and fourth generations were still modulated; although they did not cluster in a recognizable pattern ascribable to either the number of treatments or to the generations (Figure 6). Therefore, the involvement of transcription and signaling factors such as CIPK23, NIGT1;1, NIGT1;3, NIGT1;4, NFYA, MYBs, WRKYs, bHLHs in activating the expression of N-nutritional pathway for a quicker response to counteract N-limiting conditions in plants could be postulated.

The persistence of transcriptional modulation over generations was further confirmed by measuring the physiological capability of the fourth-generation plants to re-induce the ability to take up nitrate at a higher rate. Indeed, TTCT plants reached the highest uptake rate of nitrate in comparison to the other treatments (which followed the series TTCT > CTCT > TCCT = CCCT; Figure 7). This evidence suggests that the transcriptional modulation induced by N-limiting conditions during previous generations promotes a more reactive adaptation of plants to environmental changes, as N availability in the soil solution and effect is enhanced if the plants were treated twice in N-limiting conditions and disappears after two generations under N-sufficient conditions.

## 4. Materials and Methods 

### 4.1. Plant Growth 

*Arabidopsis thaliana* (ecotype Columbia) seeds were surface sterilized by immersing them in 95% ethanol for 1 min and in a solution with 2.5% NaClO and 0.1% Triton X-100 for 7 min, followed by seven rinses in sterile water. Afterwards, sterile seeds were germinated on agar medium (0.7% Phyto agar, Duchefa Biochemie, Haarlem, The Netherlands, containing nutrients: 0.5 mM KH_2_PO_4_, 0.5 mM MgSO_4_, 0.125 mM K_2_SO_4_, 0.125 mM CaCl_2_, 0.5 mM NH_4_NO_3_, 5 μM H_3_BO_3_, 0.25 μM MnSO_4_, 0.25 μM ZnSO_4_, 0.1 μM CuSO_4_, 0.005 μM Na_2_MoO_4_, pH adjusted to 5.8 with 1 M KOH) and placed in a growth chamber under controlled climatic conditions (day/night photoperiod, 8/16 h; light intensity, 220 μmol m^−2^ s^−1^; temperature day/night, 25/20 °C; relative humidity, 70%–80%). 

After 15 days, the seedlings were transferred to hydroponic conditions for 5 weeks in an aerated nutrient solution containing: 1 mM KH_2_PO_4_, 1 mM MgSO_4_, 0.25 mM K_2_SO_4_, 0.25 mM CaCl_2_, 0.5 mM NH_4_NO_3_, 10 μM H_3_BO_3_, 0.5 μM MnSO_4_, 0.5 μM ZnSO_4_, 0.2 CuSO_4_, 0.01 μM Na_2_MoO_4_ (pH adjusted to 5.8 with 1 M KOH). 

After 5 weeks, half of the plants were subjected to a seven day period of N deprivation (0 mM NH_4_NO_3_; “T” treatment), while the other plants were maintained under the same nutrient conditions as reported above (0.5 mM NH_4_NO_3_; “C” treatment). Figure 1, illustrates the complete experimental set up for all fourth generations. At the end of the sixth week of growth in hydroponic condition, root and shoot samples were collected for the physiological and molecular analyses. Some plants were preserved in hydroponic conditions for seed production under long day conditions (light day/night photoperiod, 16/8 h). For each treatment, three biological replicates were used to generate seeds for the new generation. Seeds were collected, vernalized (21 days at 4 °C) germinated and grown as described above (day/night photoperiod, 8/16 h; light intensity, 220 μmol m^−2^ s^−1^; temperature day/night, 25/20 °C; relative humidity, 70%–80%).

Elemental composition (nitrogen, N, and carbon, C, content) of root and shoots was analyzed as previously described [42] by a CHN analyzer (CHN IRMS Isoprime 100 Stable Isotope Ratio Mass Spectrometer, Elementar, Como, Italy). Root volume was estimated based on water displacement in a graduated cylinder.

### 4.2. Net High-Affinity Nitrate Uptake in Arabidopsis Plants

On the day of the experiment, nutrient solutions were renewed and supplied with 0.5 mM Ca(NO_3_)_2_ (induction); as control, no supply of nitrate was given (no-induction). After 0, 4, 6, 8, 10, and 24 h, roots of two intact seedlings were rinsed in 0.5 mM CaSO_4_ and then immersed in 12 mL of a constantly agitated solution containing 0.5 mM CaSO_4_ and 0.1 mM KNO_3_. Net uptake rate was measured as NO_3_^−^ depletion from the solution per unit of time and grams of root [43], with samples (0.05 mL) being removed for NO_3_^−^ determination every 2 min for up to 10 min, during which the uptake rate had a linear trend. Aliquots of 0.05 mL were mixed thoroughly with 0.2 mL of 5% (*w*/*v*) salicylic acid in concentrated H_2_SO_4_. After 20 min incubation at room temperature, 4.750 mL of 2 M NaOH was added. Samples were cooled to room temperature and NO_3_^−^ amounts were determined spectrophotometrically by measuring the absorbance at 410 nm. The uptake rate of nitrate was determined in Arabidopsis plants of the first, second and fourth generation.

### 4.3. RNA Extraction

Total RNA was isolated from roots of Arabidopsis plants. A pool of whole root from three individual plants was used for each sample. The RNA extractions were performed using the Invitrap Spin Plants RNA mini kit (Stratec Molecular, Berlin, Germany) according to the manufacturer’s instructions (http://www.stratec.com/). Seventy mg of tissue were homogenized in liquid nitrogen and the powder was mixed with 900 μL of DCT solution and dithiothreitol (DTT). In order to verify the absence of genomic contamination, 1 μg of total RNA was analyzed electrophoretically on 1% agarose gel. The concentration and integrity of the RNA were checked on the Qubit^®^ 2.0 Fluorometer (Life Technologies, Saint-Aubin, France) and on the Agilent 2100 Bioanalyzer system following the manufacturer’s protocol (Agilent Technologies, Santa Clara, CA, United States).

### 4.4. RNA Sequencing

Barcoded cDNA libraries were prepared for multiplex sequencing on the Illumina HiSeq2500 platform using the TruSeq™ Stranded mRNA sample preparation kit according to the manufacturer’s instructions. Twenty-four libraries were prepared from each of the three biological replicates for each treatment. To obtain sufficient material for sequencing, the libraries were amplified by PCR for 15 cycles following the recommendations of the TruSeq™ Stranded mRNA sample preparation protocol. Final elution of each library was in 30 μL of total volume. Library concentrations and quality were assessed using the Qubit 2.0 Fluorometer (Life Technologies) and the Caliper—LabChip GX/GXII (Life Sciences LTD, Runcorn, United Kingdom). Sequencing was performed on the HiSeq2500 instrument, at the Applied Genomic Institute (IGA) at Udine, Italy.

Sequence data in FastQ format were checked for their quality using FastQC software (version 0.11.3, www.bioinformatics.babraham.ac.uk/projects/fastqc/); all samples had high overall quality. Sequences were aligned onto the latest Arabidopsis Col-0 genome assembly (released November 2010) using TopHat (version 2.0.6 www.ccb.jhu.edu/software/tophat/index.shtml) [44] and BowTie (version 2.0.2 www.bowtie-bio.sourceforge.net/index.shtml) with the following parameters: -p8 -G TAIR10 annotation.gff --library-type fr-firststrand -o. The accepted_hits.bam file produced by TopHat was used in subsequent analysis steps. This binary alignment file [45] contained both spliced and unspliced read alignments. SAM tools version 0.1.19 [45] and the “NH” (number of hits) Sequence Alignment Map flag were used to separate alignments according to the number of times a read mapped onto the reference genome. Reads mapped to a unique location in the genome were used for differential expression. Differential expression analysis of RNAseq read alignment counts was performed using Cuffdiff, with Cufflinks version 2.2.0. The transcriptomic profile of T, TT, TTC and TTCC plants was compared to that of C, CC, CCC, CCCC plants, respectively. Table 1 shows the number of transcripts differentially modulated in the four comparisons (*N* = 3, *q*-value < 0.05) and all significantly modulated transcripts were defined as “up regulated” (or “down regulated”) transcripts based on their expression value: Log_2_FC > 0.5 (or <0.5, respectively). For the Venn diagram, a filtering of Log_2_FC ≥|1.00| as threshold in at least one of four comparisons was applied (Figure 5, Table 2 and Table 3).

The data were visualized and the figures were produced using MapMan software [46]. A downloadable version for local application and a servlet version are available at http://mapman.gabipd.org/web/guest/mapman-download, as well the mapping file of Arabidopsis transcriptome (mapping release AGI_TAIR9_Jan2010) and a selection of schematic maps of metabolism and cellular processes. The overview and metabolism figures in this work were prepared using version 3.6.0RC1. 

### 4.5. Real-Time RT-PCR

Total RNA (0.5 μg) was reverse-transcribed in cDNA using 100 pmol of Oligo-d(T)_23_ (Sigma Aldrich, Milano, Italy), 20 U Prime RNase Inhibitor (Eppendorf, Hamburg, Germany), 200 U of RNase H derivative of moloney murine leukemia virus (EuroClone, Pero, Italy), according to the manufacturer’s protocol, kept for 1 h at 42 °C. After RNA digestion with 1 U RNase A (USB, Cleveland, OH, USA) for 1 h at 37 °C, gene expression analyses were performed by adding 0.16 μL of the cDNA to the real-time RT-PCR complete mix, FluoCycle™ sybr green (20 μL final volume; Euroclone, Pero, Italy), in a CFX96 Touch™ Real-Time PCR Detection (Bio-Rad Laboratories, Hercules, CA, USA). Specific primers (58 °C melting temperature, Tm) were designed to generate PCR products (range 100–120 bp) with Primer3 software version 0.4.0 (Howard Hughes Medical Institute, the National Institutes of Health and National Human Genome Research Institute, www.bioinfo.ut.ee/primer3-0.4.0/) and they were synthesized by Sigma Aldrich (Milano, Italy, Table S5). The analyses of real-time results were performed using Opticon Monitor 2 software (Bio-Rad) and R (version 2.7.2; http://www.r-project.org/) with the qPCR package (version 1.1-8). Data were normalized with respect to the transcript level of the average of three housekeeping (HK) genes: Actin2 (AT3G18780), CBP20 (AT5G44200), Ubiquitin (AT5G25760; Table S5). The expression data were analyzed using 2^−ΔΔCT^ method, where ΔΔCT = (CT of target gene – CT of HK gene) _Time x_ − (CT of target gene – CT of HK gene) _Time 0_ [47].

### 4.6. Statistical Analyses

Statistical significance was determined by one-way analysis of variances (ANOVA) using Student-Newman-Keuls method for net high-affinity nitrate uptake assays taking *p*-value < 0.05 as significant (*N* = 3, *p*-value < 0.05). Statistical analyses were performed using SigmaPlot Version 12.0 software. The statistical analyses of expression data was performed as described above (*N* = 3, *q*-value < 0.05).

## 5. Conclusions

The results indicated that a priming effect occurred when Arabidopsis plants were grown in N-limiting conditions, as their progenies exhibited an enhanced ability to take up N when the stress reappeared in the following generation(s) and these plants maintained a set of modulated gene. This priming effect can be reinforced by treating the plants for two generations in N-limiting conditions, and this effect decreases with the number of generations without any nutrient limitation. Further investigation at epigenetic level are needed to characterize how N-limiting conditions can induce a memory and preserve it in future generations of plants. In conclusion, by taking advantage of transgenerational acclimation, seed production under N-limiting conditions might provide a useful tool to improve N use efficiency in crops.

## Figures and Tables

**Table 1:** Transcripts differentially modulated in the four comparisons (*N* = 3, *q*-value < 0.05).

Comparison	Up-	Down-	Total
T vs. C	3355	2354	5709
TT vs. CC	4527	2488	7015
TTC vs. CCC	1050	2025	3075
TTCC vs. CCCC	662	781	1443

**Table 2:** Selection of the most annotated transcripts presents in the Venn diagram regions A to F of Figure 5. All significantly modulated transcripts were filtered using Log_2_FC ≥ |1.00| as threshold in at least one of the comparisons (*N* = 3, *q*-value < 0.05). Red arrows, upregulated transcripts; blue arrows, downregulated transcripts; transcripts directly involved in N acquisition are shown in bold. For each region, the complete list and annotation of the genes is reported in Table S4.

Region	Regulation	T vs. C	TT vs. CC	TTC vs. CCC	TTCC vs. CCCC
*Region A*	up-		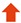	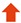	
*(42 transcripts)*	EXP12, TUB1, PDC3, CYP51G2, CKX5, ATGA2OX1, AtGolS3, UCC1, NHL12, PMSR2, PIL1, MGP, TSA1, AtRABH1e, ELIP1, MSRB7				
*Region B*	contra-		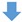	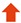	
*(85 transcripts)*	FRO4, MIR163, LSU3, GAPA-1, LBD41, AtIDD4, DDB2, APR3, F3H				
			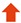	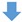	
	AtCXE17, PRP3, CSLA14, AtPP2-B5, ANAC073, IRE, ERF13, SUS6, AtSIP2, CYP702A1, sks16, ATPUP19, ATATG18D, MMP, AGC2-1, WRKY38, WRKY59, CPK22, GLR2.6, HR1, RSH2, UTR1, KT1				
*Region C*	down-		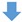	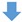	
*(36 transcripts)*	YDK1, CYP706A2, CYP71B12, BGLU45, DIC2, BT5, DREB1A, ZAT10, MYB49, PUB24, SNC1				
*Region D*	up-	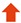	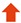	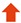	
*(116 transcripts)*	NF-YA2, TTL4, TOPP6, THAS, TGA3, SPS1, NRS/ER, NDB2, LAC1, IQD12, FUT5, FMO GS-OX4, FLS, FLA2, FLA15, DAR2, YP94B1, CYP86A4, CYP78A9, CYP705A12, CPuORF27, COR78, CER4,CAD4, B70, ST4B, OMT1, NADP-ME3, MYB53, MYB45, MYB40, MYB18, MES6, KATD, GSTU23, GST13, **GLN1;1**, EXPB1, ATEXP17, CSLC12, CSLB05, CSLA09, BAG1, ARFB1A, AGP41, AGP12, ACR6, AAP7				
*Region E*	contra-	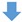	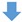	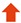	
*(191 transcripts)*	*ACO1, AN3, ALMT9, AtbZIP, MSRB5, NAS4, CLE5, CWLP, CYP705A17P,* *CYP71A16, CYP78A5, CYP81D1, CYP89A2, CYP96A12, TIP2;1, ELP, GA4, HMA2, LHCA3*, *LHCB2.1, LHCB5, PDC2, PKS2, PSAD-1, PSAL, RBCS1A, PIP2;3,ROXY2, SLAH2*				
		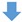	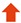	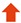 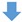	
	CAT6, ACHT5, *BGLU7*				
		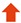	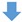	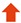	
	*GA2OX6,POP1,WAG1,RFNR1,AHP1*				
		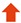	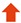	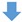	
	*AATP1, AHA7, AMT1;1, ANAC032, ANAC038, ANAC041, ANAC087, APK1B, ATH6, ATHRGP1, ATLP-3, MRP4, atnudt8, OPT7, AtPP2-A13, MYB67, BGAL8, COW1, FAR1, GAMMA-VPE, GH3.1, HHP2,LRX1, MGDC, MRH2, NSP3, PAL4, PMI1, RD21, scpl48*, *SUB, TBP1, UGT73B2, UGT73D1, WRKY58, XTR9*				
*Region F*	down-	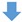	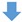	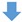	
*(103 transcripts)*	*ZYP1b, XSP1,WRKY63, UGT76E2, TEM1, CBL4, SIGE, SFP1, SAG21, RPP13, RAP2.1, PR-1-LIKE, PLP1 PLA IVA, PLL3, PAL3, OBP4, NIMIN-3, MT1C, MOT1, MEE59, MEE23, KCS3, IP5PII, CYP71B2, CYP706A7, CPuORF29, CLE6, CIPK3, CIPK23, CH1, BOR1, ATPC1, ATGSL09, ATGLR2.8, ATERF6, ATCOL5 COL5, AtbZIP58, AtbZIP3, SULTR2;2, APT2, ANAC080, ACA4, NIGT1;1, NIGT1;3, NIGT1;4*				

**Table 3:** Selection of the most annotated transcripts present in the Venn diagram regions G to N of Figure 5. All significantly modulated transcripts were filtered using Log_2_FC ≥ |1.00| as threshold in at least one of the comparisons. (*N* = 3, *q*-value < 0.05). Red arrows, upregulated transcripts; blue arrows, downregulated transcripts; transcripts directly involved in N acquisition are shown in bold. For each region, the complete list and annotation of the genes is reported in Table S4.

Region	Regulation	T vs. C	TT vs. CC	TTC vs. CCC	TTCC vs. CCCC
*Region G*	up-		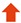	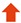	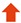
*(15 transcripts)*	*AGP7, NRT2.1, CYP71A27, XTR6, ARR7, IDL5, 2OG-Fe(II) oxygenase, RCI2B*				
*Region H*	contra-		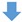	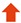	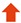
*(56 transcripts)*	*ABC tr, IPT3, TPPB, CPuORF26, AOP2, CYP71B26, APR2, LSU2, HSFA2, PIP1;4*				
			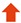	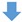	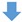
	*BMY3, UBC17, bZIP, CYP702A2, STP1, AZF2, ATG8E, ERD5*				
			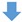	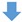	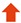
	*NIA1, CDF, EF hand*				
*Region I*	down-		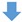	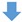	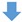
*(19 transcripts)*		*ZFP, MYB, MC9, TED6, CYP705A3, ERF104, WRKY28, WAKL4, HSPRO2*			
*Region L*	up-	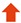	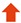	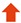	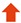
*(67 transcripts)*	*FLA16, 4CL5, JAZ4, GSTU20, UGT72E2, MYB305, YUCCA6, CYP702A5, MIR824a, TINY,* *ACR8, AtMS2, bHLH, ATSR1, CYP82F1, ATRL3, ZFP1, ATCNGC19, scpl28, ZFP5,* *XTR8, SHY2, UGT76D1, AtMYB74, CYP76C1, bZIP, MYB*				
*Region M*	contra-	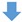	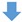	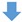	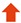
*(90 transcripts)*	*CCA1, CDF3, EXS family transporter*				
		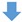	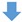	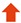	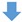
	*IRT1, CAB1, GLB1, ETR2, SUS4*				
		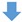	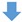	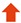	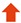
	*SOT18, ATIREG1, SLAH3, CYP735A1, SLAH1, NIC3, AtRLP24, NRT1.1, HWS, AGP30,* *NAS1, AtGDU5, VSP2, FLA13, PHI-1, MYB34, OPT, MATE*				
		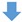	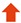	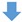	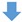
	*SEN1, NAP0, BXL1, BGAL4, MIOX2, atnudt18*				
		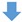	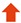	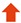	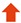 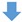
	*MES16, peroxidases, xyloglucan transferase*				
		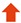	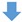	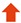	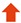
	*NIR1, G6PD3, HSP81-1, ATSDI1, HSP70, PPCK1, ATRFNR2, ATTPPA, CIPK19, PLC4*				
		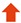	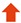	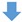	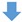
	*HO1, IQD22, LAC7*				
*Region N*	down-	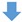	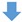	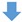	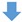
*(94 transcripts)*	*BGLU34, BARS1, BT1, BT2, UGE3, ATHB52, CAO, OXS3, AtbZIP1, MCCA, AGT1, MEE14,* *TPS9, TDT, GDH1, BGAL2, TPS10, WCRKC1, GDH2, ALDH3, NRAMP1, AtRLP9, AAE5,* *ATCTH, ASD1, EXL2, CP12-3, UGE1, ACL, CP5*				

## Supplementary Materials

Supplementary materials can be found at https://www.mdpi.com/1422-0067/20/22/5587/s1. Table S1. Significance of biological process enriched categories (GO terms) of differentially modulated transcripts in four comparisons shown in Figure 3 (N = 3, q-value < 0.05). Table S2. Enrichment analyses of gene ontology (GO) categories of up-regulated transcripts in four comparisons (N = 3, q-value < 0.05). *Onto*, ontology; *P*, biological process; *F*, molecular function; *C*, cellular component; *CM*, colorful model (the red color system means upregulated and blue means downregulated GO term). Table S3. Enrichment analyses of gene ontology (GO) categories of down-regulated transcripts in four comparisons (N = 3, q-value < 0.05). *Onto*, ontology; *P*, biological process; *F*, molecular function; *C*, cellular component; *CM*, colorful model (the red colour system means upregulated and blue means downregulated GO term). Table S4. List of modulated transcripts showed in the schematic representation in Table 2 and Table 3 and referred to Venn diagram regions A to N (Log_2_FC ≥ |1.00| in at least one of comparisons, N = 3, q-value < 0.05). Table S5. List of primer used for the real-time RT-PCR experiments. Figure S1. The fresh weight (FW) of shoots and roots and root volume of arabidopsis plants are shown. The values are means + SD, small letters refer to statistical significance (one-way ANOVA, Student-Newman-Keuls method, N = 3, p-value < 0.05). Figure S2. The nitrogen (N) and carbon (C) content and C to N ratio (C/N) are shown for Arabidopsis plants of first, second and third generations. The values are means + SD, small letters refer to statistical significance (one-way ANOVA, Student-Newman-Keuls method, N = 3, p-value < 0.05).

## Abbreviations

N	Nitrogen
NUE	Nitrogen Use Efficiency
T	Treatment (0 mM NH_4_NO_3_)
C	Control (0.5 mM NH_4_NO_3_)
