# Supplementary material for: Transgenerational Response to Nitrogen Deprivation in Arabidopsis thaliana
Source: Int J Mol Sci. 2019 Nov 8;20(22):5587. doi: 10.3390/ijms20225587 (PMC6888700; doi:10.3390/ijms20225587)

Supplementary material

**Table S1.** Significance of biological process enriched gene ontology (GO) categories (GO terms) of differentially modulated transcripts in four comparisons shown in Figure 3 (N = 3, q-value < 0.05).

|  |  |  |  | **T *vs* C** | |  | **TT *vs* CC** | |  | **TTC *vs* CCC** | |  | **TTCC *vs* CCCC** | |  |
| --- | --- | --- | --- | --- | --- | --- | --- | --- | --- | --- | --- | --- | --- | --- | --- |
|  | *GO category:* | |  | *FDR* | *Num* |  | *FDR* | *Num* |  | *FDR* | *Num* |  | *FDR* | *Num* |  |
|  | GO:0009987 | cellular process |  | 2.1e-12 | 2833 |  | 1.1e-08 | 3407 |  | 7.7e-08 | 1537 |  | 0.0059 | 709 |  |
|  | GO:0008152 | metabolic process |  | 6.8e-14 | 2705 |  | 2.3e-05 | 3163 |  | 3.6e-06 | 1438 |  | 1.1e-09 | 734 |  |
|  | GO:0044699 | single-organism process |  | 1.5e-36 | 2382 |  | 3.1e-49 | 2979 |  | 5.8e-24 | 1306 |  | 6.8e-32 | 706 |  |
|  | GO:0050896 | response to stimulus |  | 2.3e-43 | 1744 |  | 4.1e-31 | 2008 |  | 3.1e-32 | 980 |  | 3.3e-29 | 514 |  |
|  | GO:0065007 | biological regulation |  | 1.3e-05 | 1376 |  | 2.1e-05 | 1676 |  | 1.2e-10 | 822 |  | 1.6e-05 | 387 |  |
|  | GO:0050789 | regulation of biological process |  | 3.5e-06 | 1228 |  | 1.7e-06 | 1502 |  | 6.8e-11 | 736 |  | 0.00059 | 332 |  |
|  | GO:0032501 | multicellular organismal process |  | n.s. | / |  | 0.014 | 844 |  | n.s. | / |  | n.s. | / |  |
|  | GO:0032502 | developmental process |  | 0.025 | 729 |  | 0.00036 | 926 |  | 0.033 | 405 |  | 0.013 | 205 |  |
|  | GO:0051179 | localization |  | 0.03 | 577 |  | 6.7e-09 | 798 |  | 0.00018 | 348 |  | n.s. | / |  |
|  | GO:0023052 | signaling |  | 1.5e-10 | 555 |  | 1e-05 | 620 |  | 9.6e-10 | 323 |  | 0.00043 | 146 |  |
|  | GO:0040007 | growth |  | n.s. | / |  | 0.00016 | 239 |  | n.s. | / |  | n.s. | / |  |
|  | GO:0051704 | multi-organism process |  | n.s. | / |  | n.s. | / |  | 0.027 | 220 |  | 0.00087 | 122 |  |
|  | GO:0048511 | rhythmic process |  | 0.027 | 52 |  | n.s. | / |  | 0.039 | 31 |  | 5.7e-06 | 27 |  |
|  | GO:0002376 | immune system process |  | n.s. | / |  | n.s. | / |  | 0.0091 | 66 |  | n.s. | / |  |
|  |  |  |  |  |  |  |  |  |  |  |  |  |  |  |  |

**Table S2.** Enrichment analyses of gene ontology (GO) categories of up-regulated transcripts in four comparisons (N = 3, q-value < 0.05). *Onto*, ontology; *P*, biological process; *F*, molecular function; *C*, cellular component; *CM*, colourful model (red colour system means up regulated and blue means down regulated GO term).

**Table S3.** Enrichment analyses of gene ontology (GO) categories of down-regulated transcripts in four comparisons (N = 3, q-value < 0.05). *Onto*, ontology; *P*, biological process; *F*, molecular function; *C*, cellular component; *CM*, colourful model (red colour system means up regulated and blue means down regulated GO term).

**Table S4.** List of modulated transcripts showed in the schematic representation in Table 2 and Table 3 and referred to Venn diagram regions A to N (Log2FC ≥ |1.00| in at least one of comparisons, N = 3, q-value < 0.05).

**Table S5.** List of primer used for the real-time RT-PCR experiments. *AMTs*, ammonium transporters; *ANR*, MADS box transcription factor; *CIPK*, CBL-interacting protein; *DUR3*, urea transporter; *GDH*, glutamate dehydrogenase; *GLN*, glutamine synthetase; *GLT*, glutamate synthase; *GLU*, glutamate synthase; *LBD*, LOB domain-containing protein; *NIA*, nitrate reductase; *NIGTs*, nitrate-inducible GARP-type transcriptional repressors; *NRTs*, nitrate transporters.

| **AGI code** | **Symbol** | **Forward** | **Reverse** |
| --- | --- | --- | --- |
|  |  |  |  |
| AT3G18780 | *Actin2* | GCCATCCAAGCTGTTCTCTC | ACCCTCGTAGATTGGCACAG |
| AT5G44200 | *CBP20* | TACTGGCTCATTGGGAGCTT | CTCTTCCATGGCGATTTTGT |
| AT5G25760 | *Ubiquitin* | CTTGGACGCTTCAGTCTGTG | TGAACCCTCTCACATCACCA |
|  |  |  |  |
| AT3G03910 | *GDH3* | CGAGCATGCTGAAGAAAACA | CCTGTTGATGACTCCCCCTA |
| AT5G16570 | *GLN1;4* | AGGTCCTCAGGGACCGTACT | GCCGTTAGTCCCACTGACAT |
| AT5G45380 | *DUR3* | TGATCCATGCGGTGTGTAGT | TAGCTCTTCGGCAGGAACAT |
| AT3G21670 | *NRT1.3* | TGCTTACGTTGGACAGCTTG | ACTCACGAAGAATCCCATCG |
| AT5G60770 | *NRT2.4* | TTAGGGTCCACGTCCTTCAC | TACCATCAGGGAGGTCTTGC |
| AT1G12940 | *NRT2.5* | TGTTTTGTGGACCCTCTTCC | TTTCGCTAATGCTGGTTTCC |
| AT1G12110 | *NRT1.1/NPF6.3* | CAAGATGCAATCCAACAACG | ACCCGAGACACTAGCCTTCA |
| AT4G37540 | *LBD39* | ATTCTTTGGTCGTGCTGGTC | TAACCGGATTCACCGTTCTC |
| AT5G18170 | *GDH1* | ACCGAAGCTTTGCTTAACGA | CAATCTTCCCACCCTTTTCA |
| AT5G07440 | *GDH2* | ATGGGCACTAACGCTCAAAC | CACCAAGATCAATGGGCTTT |
| AT1G13300 | *NIGT1.4/HRS1* | TGGAAATCACGATCCAGACA | GTCTCCTGCTGCAACCTTTC |
| AT2G15620 | *NIR1* | AACTCGAGGAAACCGACAGA | TCCCATTTGGCATTCTCTTC |
| AT5G67420 | *LBD37* | TGCTTTGTTTCAGTCGTTGC | CAACAGCCGCTTGACAGATA |
| AT3G25790 | *NIGT1.3/HHO1* | CGAGCTTCCTCTTTGCGTAG | AGCACTCCGACTGTCCGTAT |
| AT1G77760 | *NIA1* | TGGTCGGATGGTTAAATGGT | CAGCTCAGCATCAACGAGAG |
| AT1G30270 | *CIPK23* | GCTGATGTCAGCCTTGATGA | TCGAGATGAGCTCAAAAGCA |
| AT5G53460 | *GLT1* | GGAACGAAGTCATGGAGGAA | AGAGTTCTCCTGGTGGCAGA |
| AT1G25550 | *NIGT1.1/HHO3* | GATGTTCAAGAGCGGTGACA | AGGGAGCTCACGTTGAAAGA |
| AT1G68670 | *NIGT1.2/HHO2* | GCTGGAGATGAGGAAAGACG | CGTTTAGGAATCGACGGTGT |
| AT3G49940 | *LBD38* | TCATCACTGCCGATTCTCAA | AGAGATCAAGCTCCGACGAA |
| AT2G14210 | *ANR1* | TCGATCCTTTGTGATGCAGA | CCGCTCAATGATTGTTTTCA |
| AT1G64780 | *AMT1;2* | TGCTTTGTGCTGGATCAGTC | TGCGAATCCGAAGAGGTAGT |
| AT1G08090 | *NRT2.1* | TCATCCGGGAGAATCTCAAC | GTAACGGGGACCCAAAAGAT |
| AT2G38290 | *AMT2;1* | GGGGAGGTGGGTTTCTGTAT | CTTAGGCCTTGGTCCTACCC |
| AT4G13510 | *AMT1;1* | GGTTTGCAGCCATAACTGGT | GAGCTTCTCAGCGAGCTTGT |
| AT2G41220 | *GLU2* | CCAAACACGGAACAGGTCTT | CTCAGAGGCCCAACTTTCAG |
| AT3G45060 | *NRT2.6* | TGGTCTTCCACGTCATCAAA | ATCTTGGCCGAGAGTGAGAA |
|  |  |  |  |

**Figure S1.** Fresh weight of roots, shoots and root volume of Arabidopsis plants are shown. The values are means + SD, small letters refer to statistical significance (one-way ANOVA, Student-Newman-Keuls method, N = 3, p-value < 0.05).

**
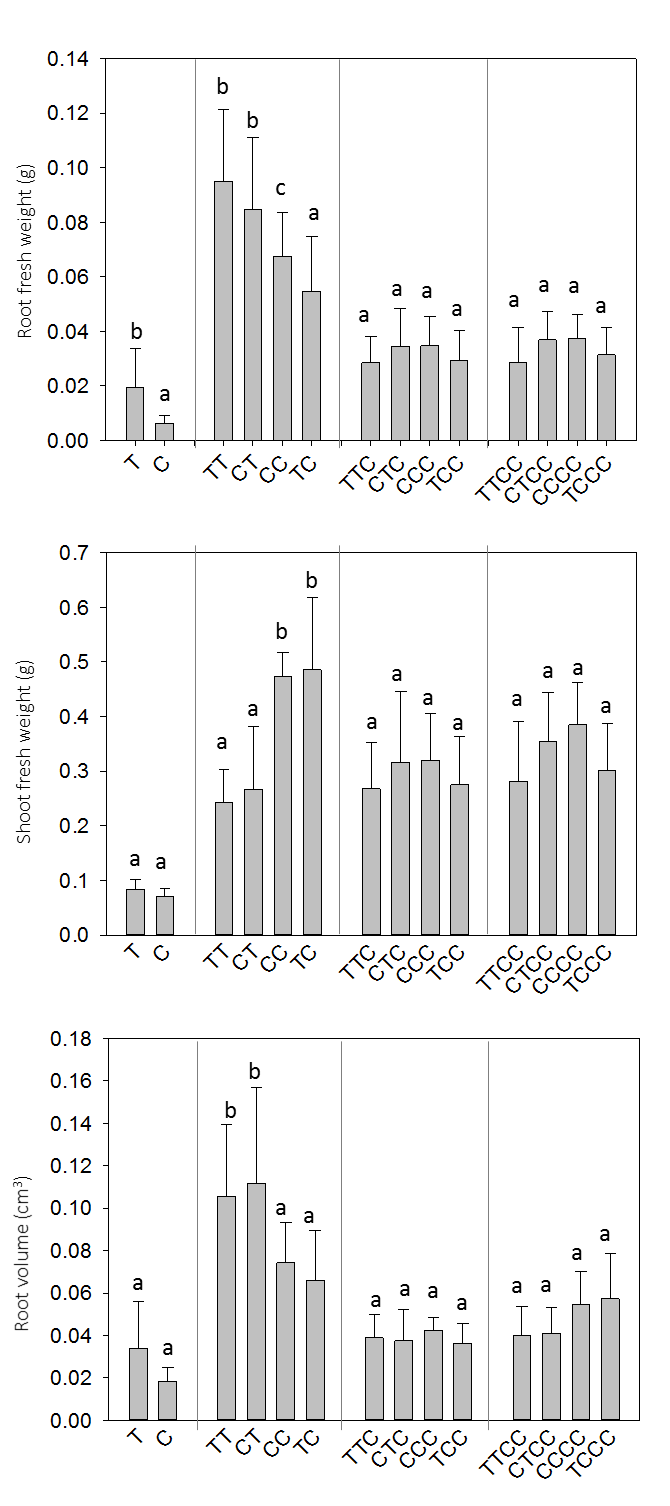
**

**Figure S2.** Nitrogen (N) and carbon (C) contents and C to N ratio (C/N) are shown for Arabidopsis plants of first, second and third generations. The values are means + SD, small letters refer to statistical significance (one-way ANOVA, Student-Newman-Keuls method, N = 3, p-value < 0.05).


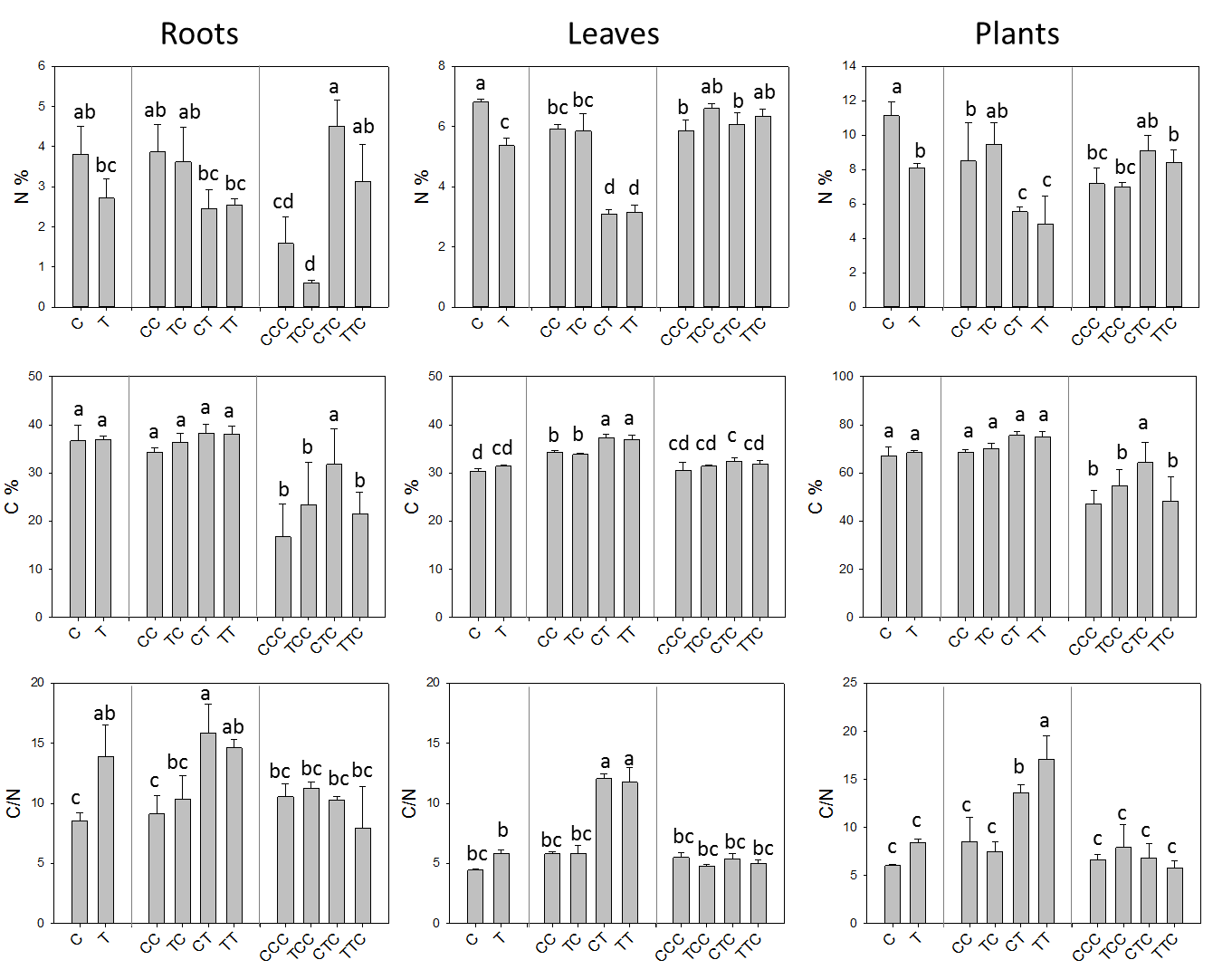

Supplement: Supplementary file 1 [file ijms-20-05587-s001.zip › Supplementary material/Supplementary material.docx]
